# Sepsis-related acute respiratory distress syndrome in children with cancer: the respiratory dynamics of a devastating condition

**DOI:** 10.5935/0103-507X.20160077

**Published:** 2016

**Authors:** Rodrigo Genaro Arduini, Orlei Ribeiro de Araujo, Dafne Cardoso Bourguignon da Silva, Andreza Almeida Senerchia, Antonio Sergio Petrilli

**Affiliations:** 1 Intensive Care Unit, Grupo de Apoio ao Adolescente e à Criança com Câncer, Instituto de Oncologia Pediátrica, Universidade Federal de São Paulo - São Paulo (SP), Brazil.; 2 Department of Clinical Research, Grupo de Apoio ao Adolescente e à Criança com Câncer, Instituto de Oncologia Pediátrica, Universidade Federal de São Paulo - São Paulo (SP), Brazil.; 3 Department of Pediatric Oncology, Grupo de Apoio ao Adolescente e à Criança com Câncer, Instituto de Oncologia Pediátrica, Universidade Federal de São Paulo - São Paulo (SP), Brazil.

**Keywords:** Respiration, artificial, Respiratory distress syndrome, adult, Sepsis, Neoplasms, Child, Respiração artificial, Síndrome do desconforto respiratório do adulto, Sepse, Neoplasias, Criança

## Abstract

**Objective:**

To evaluate the clinical course and respiratory parameters of mechanically ventilated children with cancer suffering from sepsis-related acute respiratory distress syndrome.

**Methods:**

This 2-year prospective, longitudinal, observational cohort study enrolled 29 children and adolescents. Clinical data, measurements of blood gases and ventilation parameters were collected at four different time points. Fluctuations between measurements as well as differences in estimated means were analyzed by linear mixed models in which death within 28 days from the onset of acute respiratory distress syndrome was the primary endpoint.

**Results:**

There were 17 deaths within 28 days of acute respiratory distress syndrome onset and another 7 between 29 - 60 days. Only 5 patients survived for more than 60 days. Nine (31%) patients died as a direct consequence of refractory hypoxemia, and the others died of multiple organ failure and catecholamine-refractory shock. In 66% of the measurements, the tidal volume required to obtain oxygen saturation equal to or above 90% was greater than 7mL/kg. The estimated means of dynamic compliance were low and were similar for survivors and non-survivors but with a negative slope between the first and final measurements, accompanied by a negative slope of the tidal volume for non-survivors. Non-survivors were significantly more hypoxemic, with PaO_2_/FiO_2_ ratios showing lower estimated means and a negative slope along the four measurements. Peak, expiratory and mean airway pressures showed positive slopes in the non-survivors, who also had more metabolic acidosis.

**Conclusions:**

In most of our children with cancer, sepsis and acute respiratory distress syndrome progressed with deteriorating ventilation indexes and escalating organic dysfunction, making this triad nearly fatal in children.

## Abstract (pt)

**Objetivo:**

Avaliar a evolução clínica e os parâmetros respiratórios de crianças com câncer submetidas à ventilação mecânica que apresentavam síndrome do desconforto respiratório agudo relacionada à sepse.

**Métodos:**

Este estudo longitudinal, prospectivo e observacional de coorte com duração de 2 anos incluiu 29 crianças e adolescentes. Dados clínicos, avaliações de gasometria sanguínea e parâmetros ventilatórios foram coletados em quatro momentos diferentes. As flutuações entre as avaliações e as diferenças entre as médias estimadas foram analisadas por meio de modelos lineares mistos, tendo como parâmetro primário (*endpoint*) a ocorrência de óbito dentro de 28 dias após o início da síndrome do desconforto respiratório agudo.

**Resultados:**

Ocorreram 17 óbitos dentro de 28 dias após o início da síndrome do desconforto respiratório agudo, e outros 7 entre 29 e 60 dias. Apenas cinco pacientes sobreviveram por mais de 60 dias. Nove (31%) pacientes faleceram como consequência direta de hipoxemia refratária, e os demais em razão de falência de múltiplos órgãos e choque refratário a catecolaminas. Em 66% das avaliações, o volume corrente demandado para obter saturação de oxigênio igual ou acima de 90% foi superior a 7mL/kg. As médias estimadas de complacência dinâmica foram baixas e similares para sobreviventes e não sobreviventes, porém com inclinação negativa da reta entre a primeira e última avaliações, acompanhada por uma inclinação negativa da reta para volume corrente nos não sobreviventes. Os não sobreviventes tiveram significantemente mais hipoxemia, com relações PaO_2_/FiO_2_ que demonstravam médias mais baixas e inclinação negativa da reta nas quatro avaliações. As pressões pico, expiratória e média das vias aéreas demonstraram inclinações positivas na reta para os não sobreviventes, que também apresentaram mais acidose metabólica.

**Conclusões:**

Na maioria de nossas crianças com câncer, a sepse e a síndrome do desconforto respiratório agudo evoluíram com deterioração dos índices ventilatórios e progressiva disfunção de órgãos, o que tornou esta tríade praticamente fatal em crianças.

## INTRODUCTION

Adult cancer patients with acute respiratory distress syndrome (ARDS) have a significantly higher risk of death compared with those without cancer. Additionally, these patients are more critically ill and are likely to have pneumonia and sepsis as a result of ARDS.^(1)^ Adults who develop sepsis-related ARDS present PaO_2_/FiO_2_ ratios (partial pressure of oxygen in arterial blood/fraction of inspired oxygen) that are significantly lower than those with non-sepsis-related ARDS; they also have higher mortality at 28 and 60 days, experience fewer intensive care unit (ICU)-free and ventilator-free days, and exhibit lower successful extubation rates.^(2)^ Sepsis and respiratory failure account for approximately 2/3 of hemato-oncology patients admitted to the pediatric intensive care unit (PICU),^(3)^ but little is known about the clinical course of ARDS in this group. Children with cancer who develop ARDS are extremely ill, and the mortality is unacceptably high (64.7% in one study).^(4)^

Despite efforts in basic and clinical research, ARDS mortality remains relatively unchanged. Various strategies have been attempted to revert hypoxemia, including recruitment maneuvers, ventilation modes, inhaled vasodilators and extracorporeal membrane oxygenation. Although these interventions improved oxygenation, none was able to improve mortality.^(5)^ There are no effective therapies, and clinical tests show limited success: only the prone position and the use of low tidal volumes (TV) demonstrated consistent evidence of mortality reduction.^(6)^ The prone position is usually applied without major difficulties, but the use of low TV depends on pulmonary conditions and is therefore not possible for all children with cancer, ARDS and sepsis.

Given the severity of the disease, its high mortality rate and the low number of studies available, it is relevant to improve the knowledge on this topic. The aim of this study was to evaluate the clinical course and respiratory parameters of mechanically ventilated children with cancer suffering from sepsis-related acute respiratory distress syndrome.

## METHODS

After approval by the Ethics Committee (Universidade Federal de São Paulo - UNIFESP - Nº 0031/11) and with a waiver of informed consent, this prospective, longitudinal, observational cohort enrolled 29 children with malignant diseases and sepsis-related ARDS who required mechanical ventilation for more than 24 hours and were admitted to the PICU from February 2011 to January 2013. Sepsis was defined as systemic inflammatory response syndrome caused by suspected or proven infection,^(7)^ sepsis-related ARDS was defined as ARDS developing in patients with sepsis,^(2)^ and ARDS was defined according to the European American Consensus Conference criteria. The data were retrospectively analyzed to confirm the diagnosis according to the Berlin definitions.^(8)^ No interventions or blood sample collections were performed in these patients in addition to the usual protocol for standard care. The ventilation protocol used in the ICU follows the guidelines of the III Brazilian consensus on mechanical ventilation.^(9)^

Pediatric Logistic Organ Dysfunction (PELOD) scores, white blood cell counts, values of ventilator settings and measurements (peak pressure [PP], positive end-expiratory pressure [PEEP], TV per kg and mean airway pressure [MAP]) and arterial blood gases were collected at four points: (1) at the time of endotracheal intubation; (2) at the moment of the ARDS diagnosis, (3) at the lowest PaO_2_/FiO_2_ ratio throughout the whole period of mechanic ventilation; and (4) at the last blood sample analysis before the outcome. Fixed temporal variations were not established due to the risk of death at any time of clinical course. Sex, age, weight, platelet count, hemoglobin, and coagulation tests were collected at the time of ARDS diagnosis.

The oxygenation index (OI) was calculated according to Ortiz et al.: OI = FiO_2_ x MAP/PaO_2_.^(10)^ Dynamic compliance (Cdyn) was calculated as TV/(PIP - PEEP). Normal Cdyn values are 1.1 to 2.0mL/cmH_2_O/kg in healthy infants.^(11)^

Data were analyzed with Statistical Package for Social Science (SPSS) v. 20.0 (IBM Corp., Armonk, NY, USA) and Minitab 17 (Minitab Inc., State College, PA, USA). Considering that blood gas data and ventilation parameters were obtained in repeated measurements, and these usually result in correlated errors, linear mixed models were used to evaluate fluctuations between measurements and differences in estimated means; the outcome "death within 28 days" (more likely related to ARDS) was the fixed effect. In mixed models, the intercept is the predicted value of the dependent variable when all of the independent variables are zero; thus, in our models, intercepts represent the estimated mean value of a measurement at the baseline, or first measurement. Mixed models can also handle different temporal variations between repeated measures.^(12)^ ROC curves were used to obtain cutoff values of Pa0_2_/FiO_2_ ratios and OI as predictors of survival or death within 28 days, both in the sample and in Monte Carlo simulations. A threshold for statistical significance was set at p < 0.05.

## RESULTS

There were 17 deaths within 28 days of ARDS onset and another 7 between 29 - 60 days. Only 5 patients survived for more than 60 days. Clinical and demographic data of the patients are presented in table 1. The duration of mechanical ventilation in survivors was 19 days (median, interquartile range 25^th^ - 75^th^: 14 - 25 days), with a mean ICU stay of 36.6 days and a standard deviation (SD) of 9.7 days. Only 9 (31%) patients died as a direct consequence of refractory hypoxemia. The others died of multiple organ failure and catecholamine-refractory shock, and no deaths could be directly attributed to cancer or hematologic disease in the study period. Two non-survivors had failure of two organs or systems in addition to the lungs, and the others had 3 or more failures of organs or systems. All patients received vasoactive drugs and sedation. Three patients received recruitment maneuvers, and the prone position was used in two (all non-survivors). High frequency oscillatory ventilation was not used. Non-survivors remained in the ICU for a mean of 20.4 days (SD 6.49), and the median time of invasive mechanical ventilation (IMV) was 11.5 days (interquartile range 25^th^ - 75^th^: 9 - 14.7).

**Table 1:** Demographic and clinical data

Variables	
Age (months)	120 (33 - 148)
Weight (kg)	26 (15 - 45)
Male sex	64
Underlying disease	
Hematologic	51.7
Solid tumors	48,3
Hematopoietic stem cell transplantation	6 (20.7)
Leucocyte count (/mm^3^) *	280 (2 - 2480)
Neutrophil count (/mm^3^) *	96 (6 - 2467)
Hemoglobin (g/dL) *	8.6 (7.9 - 9.9)
Platelet count (/mm^3^) *	31900 (18000 - 46200)
Activated thromboplastin time (sec) *	39 (33.6 - 49.2)
Prothrombin time (sec) *	16 (14.5 -19.2)
PaO_2_/FiO_2_ *	207 (169.7 - 245.2)
PELOD score *	11 (1 - 12)
Oxygenation index *	6.6 (4.8 - 10)

PaO_2_/FiO_2_ - partial pressure of oxygen in arterial blood/fraction of inspired oxygen; PELOD - pediatric logistic organ dysfunction. Values are listed as medians and interquartile ranges (25-75), (%) and N (%).

* at acute respiratory distress syndrome diagnosis.

Table 2 shows data for the estimated means and intercepts observed in mixed models for ventilator settings and measurements. The PaO_2_/FiO_2_ ratio showed a significant difference in the intercepts and estimated means. There was also a difference in the slope, with a steep drop (-112 in the estimated mean difference) between the initial measurement observed and that corresponding to the worst PaO_2_/FiO_2_ ratio. In a logistic regression model with the worst values of PaO_2_/FiO_2_ as predictors of death within 28 days, the observed odds ratios were 0.9793 (p = 0.022); i.e., each unit increase in the ratio corresponded to an increase of 2% in the chance of survival. Values equal to or greater than 100 showed diagnostic sensitivity of 75% for survival within 28 days as well as a specificity of 70%, with an area under the ROC curve (AUC) of 0.8; p = 0.005. In a Monte Carlo simulation with 10,000 patients in each group, constructed from a random distribution based on the values of the sample, the sensitivity was 56% with a specificity of 85% and AUC = 0.77 (p < 0.0001).

**Table 2:** Estimated means and intercepts for ventilator settings and measurements in mixed models

	Non-survivors	Survivors	p value
PaO_2_/FiO_2_ ratio			
Intercepts	151.9	267.9	0.007
Estimated means (SE)	183.6 (11.7)	225.9 (13.4)	0.02
Oxygenation index			
Intercepts	19.2	6.4	0.002
Estimated means (SE)	13.8 (1.1)	7.93 (1.2)	0.001
Dynamic compliance (mL/cmH_2_O/kg)			
Intercepts	0.45	0.65	0.031
Estimated means (SE)	0.54 (0.03)	0.62 (0.04)	0.14
Peak pressure (cmH_2_O)			
Intercepts	32.8	22.7	0.001
Estimated means (SE)	26.34 (0.9)	24.7 (1.1)	0.25
Mean airway pressure (cmH_2_O)			
Intercepts	19.2	13.9	0.019
Estimated means (SE)	16.7 (0.7)	14.5 (0.8)	0.12
Positive end-expiratory pressure (cmH_2_O)			
Intercepts	15.2	9.2	0.000
Estimated means (EP)	12.1 (0.4)	10.4 (0.5)	0.025
Tidal volume (mL/kg)			
Intercepts	7.7	8.42	0.23
Estimated means (EP)	7.97 (0.2)	8.2 (0.3)	0.5

PaO_2_/FiO_2_ - partial pressure of oxygen in arterial blood/fraction of inspired oxygen. SE - standard error.

The oxygenation index showed a significant difference in the intercepts and estimated means. The slope was positive, with a significant mean difference of +12.7 between the first and last measurement (p = 0.009) for non-survivors. One value equal to or greater than 14.5 showed diagnostic sensitivity of 69% to death within 28 days as well as specificity of 83%, with an area under the ROC curve of 0.83; p = 0.003. In a Monte Carlo simulation with 10,000 patients in each group, sensitivity was 73% with a specificity of 53% and AUC = 0.73 (p < 0.0001).

There were significant differences in the intercepts for peak pressures and MAP, but not in the estimated means. The slope was positive for PP, with a significant mean difference of +11.7cmH_2_0 between the initial and final measurements (p = 0.007) for the non-survivors. The mean difference in MAP (+6cmH_2_0 between the initial and final measurement) was not significant (p = 0.059).

**Figure 1:**
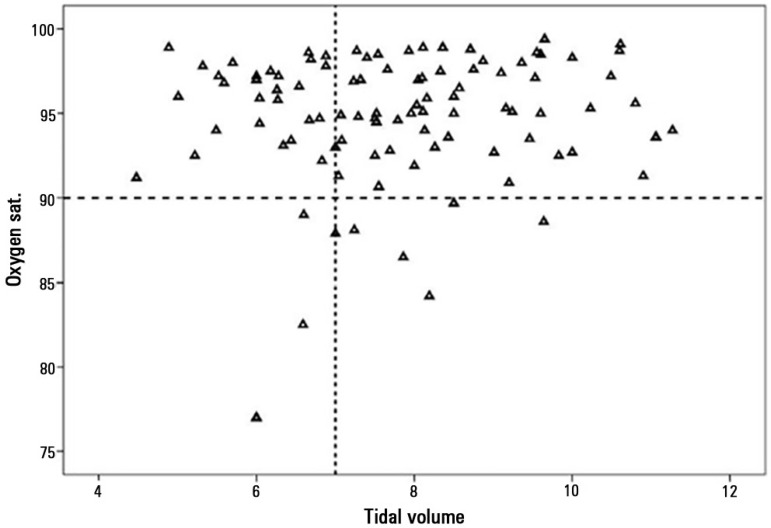
Plot of current tidal volumes per kg and concomitant oxygen saturation values.

Tidal volumes per kg were similar both in the intercepts and estimated means for non-survivors and survivors, but there was a negative slope between the initial measurement and that corresponding to the worst PaO_2_/FiO_2_ ratio (9 and 7.37, p = 0.003). Among the measurements, 55% were performed in pressure controlled ventilation, while 45% were volume controlled. Figure 1 illustrates the observation that in 66% of the measurements, the TV required to obtain oxygen saturation equal to or above 90% was greater than 7mL/kg.

**Figure 2:**
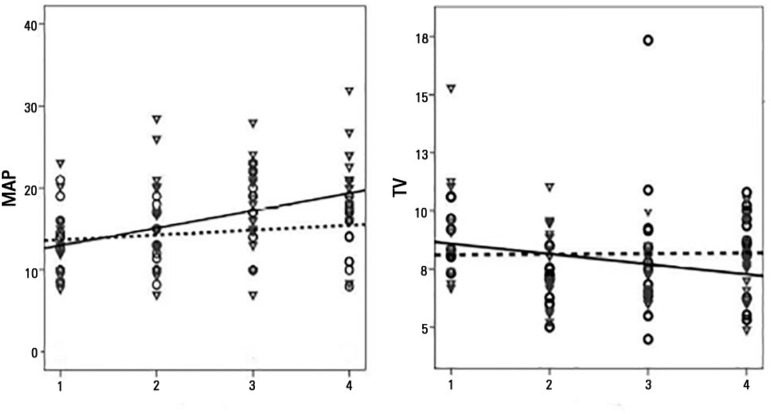
Best fit lines of the mean airway pressure and tidal volume values in non-survivors (continuous line and triangles) and survivors (dashed line and circles). The X-axis represents the four time points of observation. MAP - mean airway pressure; TV - tidal volumes.

**Figure 3:**
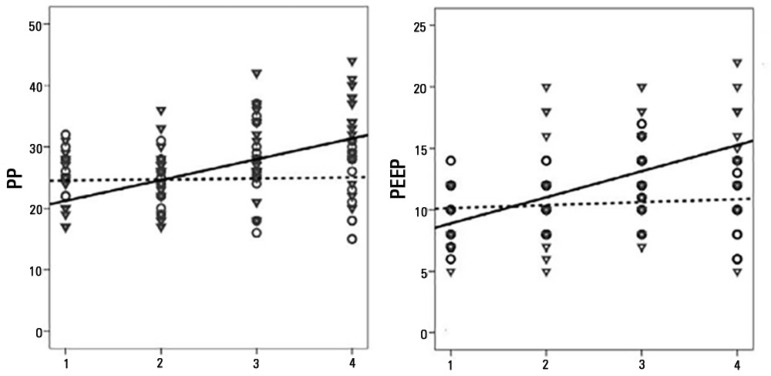
Best fit lines of peak inspiratory pressure and positive end-expiratory pressure values in non-survivors (continuous line and triangles) and survivors (dashed line and circles). PP - peak inspiratory pressure; PEEP - positive end-expiratory pressure.

**Figure 4:**
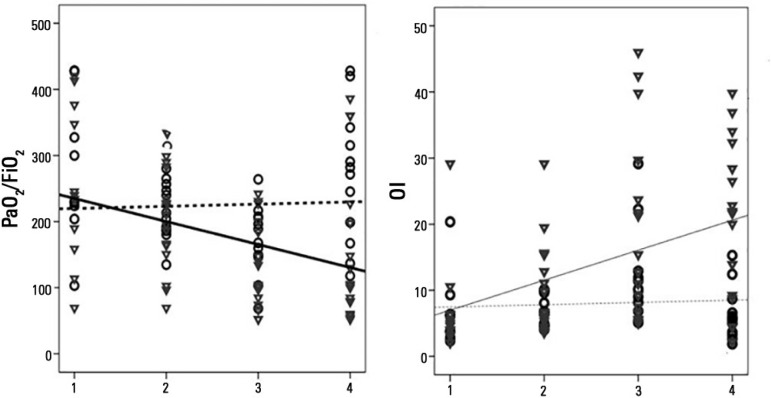
Best fit lines of partial pressure of oxygen in arterial blood/fraction of inspired oxygen as well as oxygenation index values in non-survivors (continuous line and triangles) and survivors (dashed line and circles). PaO_2_/FiO_2_ - partial pressure of oxygen in arterial blood/fraction of inspired oxygen; OI - oxygenation index.

There was a positive slope (mean difference of +3.13cmH_2_0, p = 0.002) between the first and final PEEP measurement for the non-survivors. Figures 2 to 4 show plots of ventilator parameters and oxygenation indices. There was no significant fluctuation in inspiratory times.

Estimated means of dynamic compliance were low and were similar for survivors and non-survivors, but there was a difference in the intercepts and a negative slope between the first and final measurements in the non-survivors (-0,26mL/cmH_2_O/kg, p = 0.02).

Blood gas analysis revealed that patients who died showed a trend of metabolic acidosis, with a difference in the intercepts for pH between survivors and non-survivors (7.37 and 7.30, p = 0.005) and with estimated means of 7.40 (standard error (SE) 0.02) and 7.32 (SE 0.02, p = 0.005). The mean difference between initial measurement and that corresponding to the worst PaO_2_/FiO_2_ ratio was -0.087 (p = 0.02). For bicarbonate, the intercepts were 25.5 and 21.6 for survivors and non-survivors, respectively, with estimated means of 25.9 (SE 0.9) and 22 (SE 0.8, p = 0.002). For the base excess, the intercepts were 1.71 and -2.6, with estimated means of 1.6 (SE 1.1) and -2.71 (SE 0.9, p = 0.002).

The partial pressure of carbon dioxide presented similar estimated means in survivors (42.5mmHg, SE 1.4) and non-survivors (42.6, SE 1.6), with a positive slope between the initial measurement and that corresponding to the worst PaO_2_/FiO_2_ ratio (mean difference +13.9mmHg, p < 0.0001). Hypercapnia was observed in 22% of the measurements in 14 patients. The PaO_2_ also showed similar means between survivors (89mmHg, SE 3.7) and non-survivors (88, SE 3.2), with a strong negative slope between the first measurement and the one corresponding to the worst PaO_2_/FiO_2_ ratio (-31.4mmHg, p < 0.0001). The oxygen saturation levels were also similar in their intercepts (95.4% and 94.5), with a negative slope between the initial and the worst measurement (-4.27, p < 0.0001).

The total number of leukocytes was also similar in the estimated means for survivors (5149/mm^3^, SE 1137) and non-survivors (4025, SE 836, p = 0.37). The estimated means of neutrophils were 3554/mm^3^ (SE 1190) and 2459 (SE 1025, p = 0.17), with a positive slope for neutrophils between the first measurement and that corresponding to the worst PaO_2_/FiO_2_ ratio (mean difference: +2843, p = 0.042). There were no significant differences in the intercepts and slope for total leukocytes count.

The PELOD score showed a difference in the intercepts (16.7 in non-survivors within 28 days and 7 in survivors (p = 0.005)). The estimated means were also different for non-survivors (13.2, SE 1.3) and survivors (7.5, SE 1.6, p = 0.037), with a positive slope for non-survivors and a mean difference of +6.94 between the first and last measurements (p = 0.01).

## DISCUSSION

Most of our patients progressed with escalating losses in organ function along with the deterioration of both oxygenation and lung compliance, reflected in the increasing demand for higher pressures and worsening of ventilation indexes. Despite the fact that only a minority of patients died as a direct consequence of respiratory failure, the authors cannot minimize the role that it played in the dying process; data show that the deterioration of gas exchange was able to discriminate patients who would die within 28 days, with some sensitivity and specificity. Although the study design does not enable conclusions about causality, it seems fair to say, based on the strength of association, that respiratory failure was a key part of this process. Ben-Abraham et al. studied 17 children with ARDS and hematological malignancies admitted to the PICU and placed under IMV. Significant differences were observed between survivors and non-survivors after the third day of hospitalization when comparing PP, PEEP and ventilation index values.^(4)^

Despite the great depletion in granulocytes, lymphocytes and monocytes, patients receiving chemotherapy are able to maintain elevated levels of inflammatory cytokines in sepsis, particularly interleukins 6 and 8;^(13)^ this suggests that production and excretion by macrophages and dendritic cells are preserved. By receiving almost the totality of cardiac output, lungs are exposed to a great number of inflammatory mediators secreted by these cells in peripheral organs, in addition to the local production by alveolar macrophages and activated endothelial cells.^(14)^ Most of our patients were neutropenic at the time of ARDS diagnosis, with a median of 96 neutrophils, which seems to demonstrate that pulmonary inflammatory events can be initiated without the participation of these cells. It is worth noting that in subsequent days, increased neutrophil counts coincided with the worst PaO_2_/FiO_2_ ratio values observed.

The hallmark of ARDS injury is alveolar inflammation, with influx of protein-rich fluid and surfactant inactivation. Compliance reduction is a consequence of alveolar collapse and subsequent exclusion of poorly aerated areas from the gas exchange. In this situation, small TV can cause a dramatic rise in airway pressure. We observed a pronounced negative slope in TV in our non-surviving patients, reflecting the progressively worse compliance. Parenchymal injury is diffuse, but not uniform, and normal areas can be present among cysts and consolidations. Elevated TV and peak pressures can promote overdistension of these normal areas, with subsequent inflammatory injury, this time induced by mechanical ventilation and similar to ARDS.^(15)^

In addition to aggravating lung injury, mechanical ventilation can also result in hemodynamic imbalance that can lead to the development of multiple organ failure.^(16)^ By means of more subtle mechanisms, decompartmentalization of the inflammatory response can be promoted, increasing alveolar-vascular permeability or opening micro-fissures, "spreading" mediators into the bloodstream, and carrying the injury from lungs to distal organs. IMV also upregulates pulmonary cytokine production: transmembrane receptors (such as integrins), stretch-activated ion-channels and the cytoskeleton are structures capable of transducing mechanical stimuli ("mechanosensing") and initiating intracellular processes. Direct trauma to the membrane of alveolar cells as well as loss of cell integrity leads to the release of intracellular cytokines to the interstitium. These mechanisms of injury involving inflammatory pathways, in which cytokines play a key role, have been termed biotrauma.^(17)^ Not only mediators are translocated but also lipopolysaccharides and bacteria.^(18)^

Immunosuppression has been recognized as a key pathophysiological mechanism in sepsis.^(19)^ In children with cancer, this mechanism is an addition to immunosuppression of the disease itself and to the depletion of immune system cells, contributing to a somber prognosis.

A possible confounding variable in this study is the fact that all patients received multiple transfusions due to anemia and thrombocytopenia. Transfusion-related acute lung injury, whose pathogenesis is related to the infusion of donor antibodies that recognize leukocyte antigens in the transfused host, or to the infusion of lipids and other biological response modifiers that accumulate during storage or processing of blood components, could act synergistically with other risk factors for acute lung injury; it could also overlap with ARDS. Even if there were a temporal relationship between transfusion and a new episode of hypoxemia, it would probably be attributed to a worsening of ARDS.^(20)^ An interesting line of research could evaluate whether a less liberal transfusion policy has an impact on this group of patients.

A less aggressive ventilation strategy based on low TV (5 - 7mL/kg), with plateau pressures lower than or equal to 30cmH_2_O, has been effective in reducing mortality in adults with ARDS.^(21)^ It also leads to a reduction in the inflammatory response not only in the lung but also in plasma, confirming that the systemic dissemination of the events originated in the lungs.^(15)^ Unfortunately, this protective strategy is based on maintaining oxygen saturation in the lower limit of normal (approximately 90%); as our data show, to maintain these saturation levels the majority of our patients required TV greater than 7mL/kg due to low lung compliance. We believe that mechanical ventilation contributed to the worsening of lung injury and high mortality. Due to the severity of the condition, we believe that aggressive measures should be attempted in order to lower mortality to a certain degree. A strategy combining permissive hypoxemia and supranormal cardiac output (by optimizing the preload and vasoactive drugs) could meet the tissue oxygen consumption demand without the burden of increasing PaO_2_.^(22)^ Recruitment maneuvers have limited application in our patients due to thrombocytopenia with the risk of pulmonary bleeding, and due to severe hemodynamic instability. Risk of bleeding is a theoretical concern, because these maneuvers have caused ultrastructural damage with detachment of the alveolar epithelium in animal models.^(23)^ However, we have no evidence from evaluations of this complication in human studies, particularly in cancer patients. Hypotension is a common complication.^(24)^ The best method to perform these maneuvers has not been defined, and this is also a limitation.^(25)^ The fact that mean values of PCO_2_ were normal in our sample illustrates the tendency to over-correct the hypercapnia, despite the recommendation to permit it. The scarce use of the prone position in our patients can be attributed to severe hemodynamic instability and a high dependency on airway and vascular access.^(26)^

This study was limited because it was single-center and observational, and involved a small sample. However, we believe it is important to show the darker side of a probably frequent clinical condition in the pediatric oncology ICU that is rarely studied. Efforts should be made to better understand ARDS in the context of sepsis in children with cancer.

## CONCLUSION

Most of our children with cancer, sepsis and acute respiratory distress syndrome progressed with deterioration in ventilation indexes accompanied by catastrophic organic dysfunction, making this triad nearly fatal in children who required mechanical ventilation. Protective ventilation strategies could be hindered by the difficulty of maintaining acceptable oxygenation with tidal volumes lower than 7mL/kg.
